# Intensity of *Nosema ceranae* infection is associated with specific honey bee gut bacteria and weakly associated with gut microbiome structure

**DOI:** 10.1038/s41598-019-40347-6

**Published:** 2019-03-07

**Authors:** Andrey Rubanov, Kaleigh A. Russell, Jason A. Rothman, James C. Nieh, Quinn S. McFrederick

**Affiliations:** 1 UCSD Division of Biological Sciences Section of Ecology, Behavior, and Evolution 9500 Gilman Drive, MC0116, La Jolla, CA 92093-0116 USA; 2 Department of Entomology, University of California, Riverside, 900 University Avenue, Riverside, CA 92521 USA; 3 Graduate Program in Microbiology, University of California, Riverside, 900 University Avenue, Riverside, CA 92521 USA

## Abstract

The honey bee, *Apis mellifera*, pollinates a wide variety of essential crops in numerous ecosystems around the world but faces many modern challenges. Among these, the microsporidian pathogen *Nosema ceranae* is one of the primary detriments to honey bee health. *Nosema* infects the honey bee gut, which harbors a highly specific, coevolved microbiota heavily involved in bee immune function and nutrition. Here, we extend previous work investigating interactions between the honey bee gut microbiome and *N*. *ceranae* by studying experimentally infected bees that were returned to their colonies and sampled 5, 10, and 21 days post-infection. We measured *Nosema* load with quantitative PCR and characterized microbiota with 16S rRNA gene amplicon sequencing. We found significant colony level variation in infection levels, and subtle differences between the microbiota of colonies with high infection levels versus those with low infection levels. Two exact sequence variants of *Gilliamella*, a core gut symbiont that has previously been associated with gut dysbiosis, were significantly more abundant in bees from colonies with high *Nosema* loads versus those with low *Nosema* loads. These bacteria deserve further study to determine if they facilitate more intense infection by *Nosema ceranae*.

## Introduction

Flower-visiting pollinators, primarily bees (such as *Apis mellifera*) and other insects^1^, are estimated to be responsible for approximately 35% of global food production directly consumed by humans^2,3^. U.S. honey bee pollination is estimated to be worth between $12.3 to $16.4 billion^4^, with crops like California almonds relying primarily on honey bee pollinators^1^. From 2006–2014, winter colony losses have been approximately 30% in the USA^5^, an unsettling trend also observed in other countries^6^. While the cause for the recent increase in colony losses has not been fully elucidated, it is partially attributed to *Nosema ceranae*, exposure to agricultural pesticides, environmental variation, and the synergistic effects between these factors^7^.

*Nosema ceranae*, originally a parasite of the Asian honey bee *Apis cerana*, underwent a host shift to infect *A*. *mellifera* and has rapidly replaced *Nosema apis* as the primary honey bee parasite worldwide^8–10^. *Nosema ceranae* reduces the number of foragers in a colony, degenerates digestive tissue, and results in an overall reduction of colony strength, particularly in combination with other factors detrimental to honey bee health^11–14^. *Nosema* solely infects the bee midgut, inhibiting genes involved in tissue renewal, leading to malnutrition and, in certain studies, increased mortality^15–17^. The virulence of *N*. *ceranae* in *A*. *mellifera*, however, varies widely, and the underlying causes of this variation are still open to debate^18^. Several studies have suggested that colonies differ in their susceptibility to *Nosema* infection^19^. However, separate research in the USA^20^ and Spain^21^ failed to support any link between *N*. *ceranae* infection and host genetics. Therefore, colony-level variation in *N*. *ceranae* susceptibility and the contribution of the microbiota to this variability remains an open question.

Surveys of *A*. *mellifera* gut microbes from four different continents have revealed that honey bee guts harbor representatives of 5–8 bacterial phylotypes involved in host nutrition, toxicity, immune function, and possibly pathogen resistance^22–25^. Interestingly, this microbiota is highly conserved despite distinct differences in environment, geography, and bee sub-species. Honey bee-specific microbes are most abundant in the hindgut, but can also be found in appreciable numbers in the midgut^26^, the sole location of *Nosema* infection^16^. The microbiota and *Nosema* interaction may account for some of the variation in responses to infection and could be manipulatable to improve bee resistance to *Nosema*.

Understanding how *Nosema* infection alters the gut microbiota has implications for developing ways to restore a healthy gut community in infected bees and, perhaps, to develop microbial treatments which combat the effects of infection. The microbiota may interact with gut pathogens in multiple ways. The honey bee microbiota stimulates expression of host antimicrobial peptides^27^, which may prime the honey bee to fight off *Nosema* infection. Other possible defensive mechanisms include competition for resources between commensal microbes and gut pathogens, secretion of chemicals that inhibit or kill pathogens, and creation of physical barriers (i.e. biofilms) on host gut epithelia^28,29^.

Short generation times allow the microbiota to respond to selection more rapidly than the bee host, and engineering the microbiota can be a rapid manner to increase host health^30^. Here, we aim to better understand whether and how the microbiota might interact with *Nosema* to add to the long-term goal of increasing honey bee health. First, we examined variation in disease load, at a colony level, to workers deliberately infected with *N*. *ceranae*. Next, we determined whether specific microbes increase or decrease in abundance with *Nosema* infection. Finally, we tested for differences in the microbiota of bees from colonies challenged with the same *Nosema* inocula, but which exhibited opposite intensities of infection. We aimed to determine if specific bacteria correlated with *N*. *ceranae* infection are targetable predictive markers of colony health for future studies.

## Results

### Nosema infection

**Figure 1:**
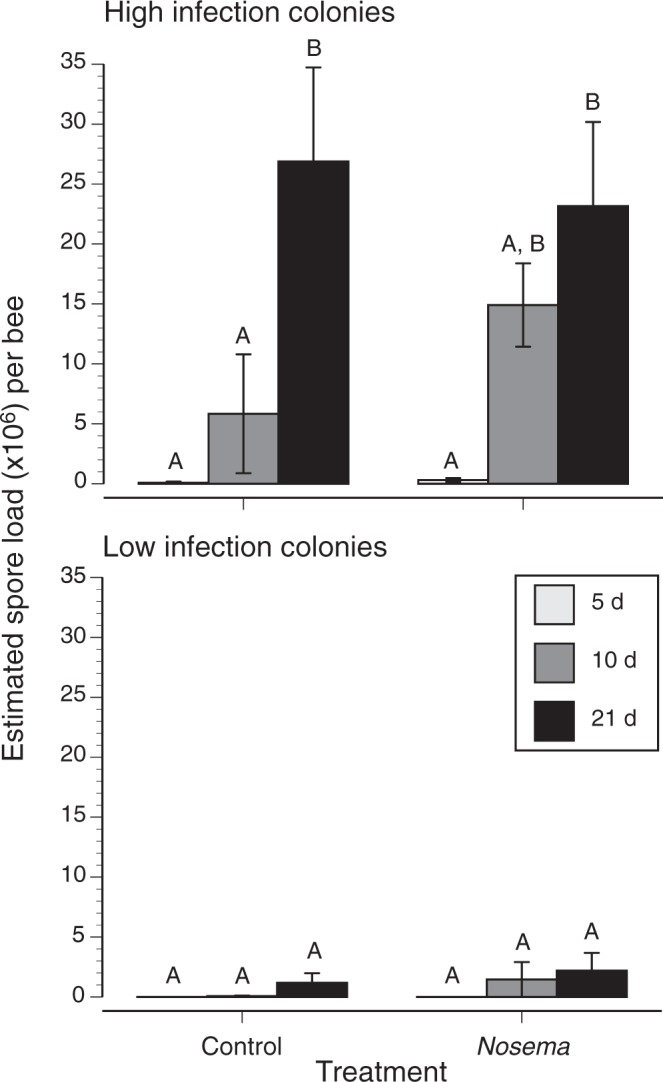
Effects of colony identity, treatment, and bee age (5, 10, or 21 day old adults) on estimated *N*. *ceranae* spore loads. Colonies are divided into two groups based upon bee infection levels (high = 3 colonies, N = 66 and low = 6 colonies, N = 82). Different letters indicate significant differences (Tukey HSD tests, *P* < 0.05).

As expected, bees became significantly more infected over time (*F*_2,136_ = 8.254, *P* < 0.0004, Fig. 1). Moreover, there was a significant colony effect (*F*_8,136_ = 11.84, *P* < 0.0001). In the same ANOVA, treatment was nearly significant (*F*_1,136_ = 3.724, *P* = 0.055, Fig. 1). We found two colony groupings with respect to *Nosema* infection load (Fig. 1). Highly infected colonies (3 colonies) had bees with significantly more estimated spores than bees in low infection colonies (6 colonies, Tukey HSD test, *P* < 0.05).

**Figure 2:**
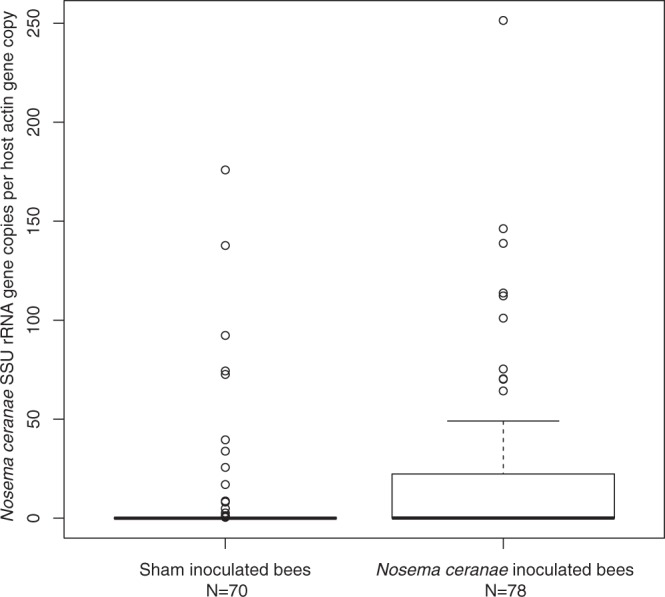
Pooled estimated spore loads (relativized to host Actin counts) for sham inoculated vs. *Nosema* inoculated bees over all colonies. While most control bees were infected with *Nosema*, given that they lived and interacted in the same colonies, the treated bees had significantly higher spore counts (more than twice as high as control bees, (*t* on ranked data = −2.478, df = 146, *P* = 0.014). Boxplots are shown.

Initially (5 days post-inoculation), controls and bees fed live *Nosema* spores had similar, low infection levels (Fig. 1). By day 21 infection levels significantly increased in the high infection colonies regardless of treatment (Tukey HSD, *P* < 0.02, Fig. 1), but did not significantly differ from original levels in the low infection colonies (Fig. 1). When all samples are considered, however, bees that were fed *Nosema* spores had significantly higher infection loads as measured by qPCR when compared to controls fed solely sucrose (Fig. 2, *t* on ranked data = −2.478, df = 146, *P* = 0.014). Despite infection in some control bees, likely due to interactions with treated bees in the colony, the average infection load in control bees was half that of treated bees.

#### Microbiota

After quality filtering, removal of sequences that were assigned to chloroplast or mitochondria (from either pollen in the bee’s gut or host mitochondria), removal of sequences that occurred in no-template control blank samples (buffer contaminants *Shewanella* and *Halomonas* and human-associated *Propionibacterium*), and removal of samples for which host actin qPCR was below our detection threshold, we retained 1,770,123 sequences across 148 individual bee samples. Based on the number of sequences per sample and the sampling depth at which rarefaction curves leveled off, we rarified each sample to 2,500 sequences, which allowed us to include 144 samples in our downstream analyses. This rarified matrix was used for all alpha and beta diversity analyses, either in its entirety or as two subsets of the entire matrix broken apart by treatment or control bees.

**Figure 3:**
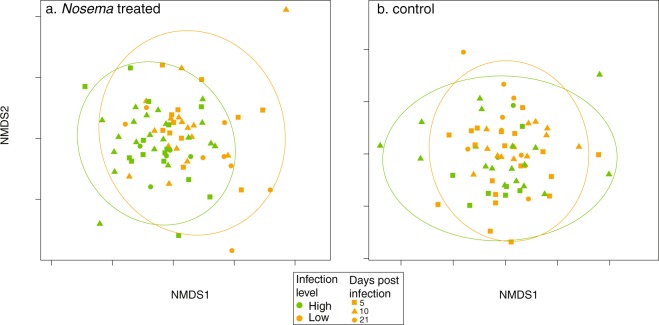
NMDS ordination coded by whether the colony of origin of the bee had high or low *Nosema* infection (color) and time points (shape). Ellipses represent 95% confidence intervals for colony infection level. There are subtle but significant differences in the composition of the gut microbiota in *Nosema* treated bees (**a**, *F*_1,70_ = 2.28, *P* < 0.01) but not in control bees (**b**, *F*_1,62_ = 1.53, *P* < 0.057).

The presence of *N*. *ceranae* in control bees may explain why Adonis analysis (permutational MANOVA) of Bray-Curtis distances of gut microbiota composition showed no significant difference in control versus infected bees (Fig. S1, *F*_1,142_ = 0.91, *P* = 0.56). Alpha diversity (observed ESVs) approached significance, but did not differ by treatment (Kruskal-Wallis *H*_134_ = 3.54, *P* = 0.059). To account for infection in control bees, we tested for differences in the gut microbiota of bees from colonies that were classified as having low or high *Nosema* infection (as described in the *Nosema* infection section above). Initial graphing of an NMDS ordination of Bray-Curtis distances identified one outlier sample, which we removed for clarity of visualization in Fig. 3. Inclusion or exclusion of this sample did not affect the results. First, we repeated alpha (observed ESVs) and beta diversity analyses (Bray-Curtis distances) using only *Nosema* treated bees. For *Nosema* treated samples, high infection level colonies exhibited more ESVs per sample compared to low infection colonies (Fig. S2, Kruskal-Wallis *H*_71_ = 12.07, *P* < 0.0006) and beta diversity differed between colonies with high and low infection levels (Fig. 3, *F*_1,70_ = 2.28, *P* < 0.01). Next, when we considered only control bees alpha diversity did not differ between high and low infection level colonies (Fig. S2, Kruskal-Wallis *H*_64_ = 0.12, *P* = 0.75) and beta-diversity approached significance but no longer differed between colonies with low and high infection levels (Fig. 3, *F*_1,62_ = 1.53, *P* < 0.057).

**Figure 4:**
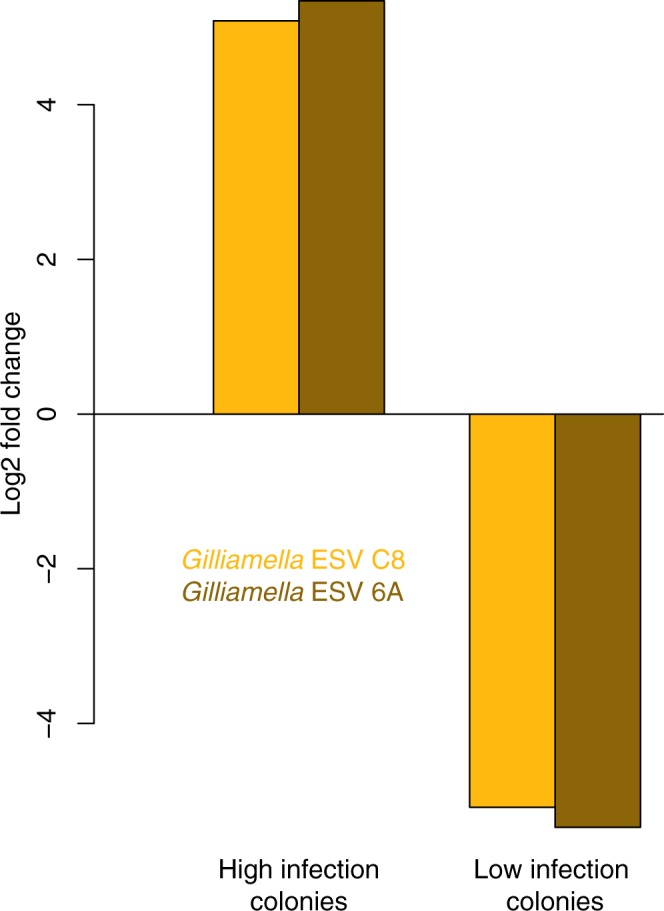
Differential abundance (log2 fold) of two *Gilliamella* ESVs in colonies with high infection levels versus colonies with low infection (high = 3 colonies, N = 66 and low = 6 colonies, N = 82). Ancom analysis identified these two ESVs as being significantly different across colonies with different infection statuses (False Discovery Rate adjusted P < 0.05).

An Ancom test to determine the differential abundance of microbes between colonies with low and high infection levels identified two ESVs that were significantly associated with infection. According to BLAST searches (max scores of 612 and 616 and sequence identities of 100%) and Naive Bayesian Classifier analyses, both ESVs were strains of the core gut symbiont *Gilliamella*. Both ESVs were positively associated with increasing *N*. *ceranae* abundance (Fig. 4). These two ESVs were also differentially abundant when only *Nosema* treated samples were considered, but not when only control bees were analyzed.

## Discussion

The intensity of *Nosema ceranae* infection has only subtle effects on the overall composition of the honey bee gut microbiome. Underlying this compositional difference are two *Gilliamella* ESVs that were significantly associated with *Nosema ceranae* load. These *Gilliamella* ESVs are members of the core honey bee microbiota and are thus likely important for host health^23,24^.

Other studies have explored interactions between *N*. *ceranae* and gut microbes by either manipulating the presence of specific strains or sequencing the microbiome of infected or uninfected bees. Corby-Harris *et al*.^31^ and Baffoni^32^ found that *Parasaccharibacter apium* and lactobacilli and bifidobacteria supplemental feedings, respectively, lowered the intensity of *N*. *ceranae* infection. Li *et al*.^33^ used a cage experiment to show that *N*. *ceranae* challenge did not affect the microbiota, but that antibiotic treatment made honey bees more susceptible to *N*. *ceranae* challenge. Hubert *et al*.^34^ used naturally occurring infection (detected by PCR) to determine that *N*. *ceranae* infected bees did not harbor significantly different microbiota compared to uninfected bees.

Our data agree with these previous studies that show that *N*. *ceranae* infection does not cause large compositional changes to the gut microbiota^34^. In contrast to these studies, which characterized the microbiota of entire bees, we dissected out and extracted DNA from midguts and hindguts only. While this allowed us to focus on the gut, future research focusing only on the midgut, where *N*. *ceranae* localizes^35^, may be promising. The midgut harbors some bacteria near the pylorus, but the honey bee rectum harbors the bulk of the gut microbiota^26^. The different localization of *N*. *ceranae* and the bulk of the microbiota may explain why no large changes in the entire gut microbiota co-occur with *N*. *ceranae* infection.

Our study also differs from these previous studies in that we returned the inoculated and control bees to their colony of origin post-inoculation. Although returning the treated bees to the colony resulted in *N*. *ceranae* exposure in our control bees, we were able to control for this exposure by quantifying *N*. *ceranae* infection intensity and determining which colonies had high and low levels of infection. Harboring treated bees in the colony allowed us to maintain field-realistic conditions for our study and control for potential colony-level differences. We are therefore able to conclude that *N*. *ceranae* infection subtly alters the overall composition of the gut microbiota in a colony setting. This paves the way for a future lab-based study that tests the effects of *Nosema* feeding on caged bees. This experiment should eliminate infection of control bees and allow us to confirm the microbiome results obtained in our current findings.

We also explicitly tested for a colony effect on *Nosema* susceptibility. Our results demonstrated that colonies significantly varied in their susceptibility to *Nosema* infection despite containing similar number of workers and being each exposed to the same number of *Nosema*-fed workers, each inoculated with the same dose of live spores. Colonies were initially uninfected, based upon standardized random sampling and mid-gut spore counts of 20 foragers per colony^36^. Gut microbiota alpha and beta diversity differed significantly, but subtly, between colonies with low or high infection levels. Furthermore, two *Gilliamella* ESVs were differentially more abundant in colonies with high infection levels. The microbiota may therefore play some role in *Nosema* infection, but it is likely that a substantial amount of variation in *Nosema* susceptibility at the colony level is due to host genetics. Future work investigating host population genomics, polyandry^37^, and *Nosema* susceptibility may therefore be promising.

*Gilliamella* is a member of the honey bee “core gut microbiome”^38^ and specific strains can degrade sugars that are found in floral nectar and are toxic to honey bees^24^. Within the honey bee-associated *Gilliamella*, however, a vast diversity of strains exists with different genomic repertoires and possible functions^22^ including a possible role in gut dysbiosis^39^. Indeed, gut dysbiosis in worker honey bees has been associated with increased *Gilliamella* counts, although one study found that *Frischella perrara* and not *Gilliamella* was significantly associated with *Nosema* infection^40^. Our finding that two specific *Gilliamella* strains are positively associated with *Nosema* infection intensity is therefore not unprecedented. The correlations between these strains and *Nosema* merit further study. For example, whether these ESVs reside in the midgut and affect the function of the protective peritrophic matrix or whether they are found in the ileum along with most gut bacteria and therefore interact with *Nosema* via the host immune system would provide insight into how these ESVs may help increase *Nosema* loads.

Because the pathogen and the majority of the bacteria studied here reside in different parts of the honey bee gut, it is perhaps not surprising that *Nosema* infection and gut microbiome composition are not strongly associated. Instead, subtle differences between colonies with high or low levels of infection allowed us to identify two *Gilliamella* ESVs that positively associate with *Nosema* infection intensity. We are therefore closer to identifying microbes that contribute to nosemosis. To be able to protect honey bees from this pathogen, we need further work to identify whether this correlation is indeed causative, and whether these bacteria can be selectively removed to promote a healthy and resistant microbiome.

## Methods

### Generating *N*. *ceranae* stock

To generate *N*. *ceranae* spore stock, we placed 25 newly emerged worker bees into a cage equipped with a syringe containing 5 ml of 2.0 M sucrose solution mixed with 1 million freshly extracted *N*. *ceranae* spores (40,000 per bee). Bees were fed only 2.0 M sucrose *ad libitum* after this 5 ml was consumed and were given 10–12 days to develop heavy infections^36^. Spores were harvested within 12 h before each new trial and stored at 4 °C to ensure viability^36^. Spores were extracted by dissecting out the midgut of infected bees (workers fed approximately 100,000 spores each for 12 days). Three midguts were placed per 100 µl DD H_2_O in a 1.5 ml Eppendorf tube, ground with Kimble polypropylene pestles, diluted to 1 ml, vacuum filtered through a Buchner funnel lined with Fisherbrand P8 Filter paper, and concentrated by centrifuging for 15 min at 10,000 rpm. The supernatant was discarded, and the precipitates were pooled and re-suspended in 500 µl DD H_2_O (modified from^41^). A hemocytometer was observed at 400x total magnification under a compound light microscope to determine spore concentrations^36^.

### Obtaining bees

Workers of *A*. *mellifera ligustica* (Spinola, 1806) bees were obtained from nine colonies (each with approximately 20,000 bees, based upon visual estimation) that were all re-queened in May 2015. The colonies were housed at UCSD’s Biology Field Station and Elliot Chaparral Reserve from June 2016 to June 2017. Colonies were initially uninfected, based upon standardized random sampling and mid-gut spore counts of 20 foragers per colony^36^. For each colony, we collected comb frames with at least 200 capped brood cells, removed all adult bees, and transferred the frames into a nuc box that was placed into incubators at 33 °C and 60–80% relative humidity. Frames were kept in incubators for no longer than 5 days and checked daily for newly emerged worker bees.

### Bee treatments

Within 24 h of emergence, 100 newly emerged workers per colony were painted on their thoraces with one of two colors of non-toxic enamel paint. Each bee was placed inside its own plastic vial into which a pipette with the treatment was inserted. Newly emerged bees are often reluctant to feed, and thus the vials were placed into racks in which strips of ultraviolet led diodes (SMD 3528, 240 lumens/m, 395–405 nm) illuminated the pipettes, attracting the bees and encouraging feeding. The majority of bees consumed the treatment in less than 1 h, as determined by visual inspection of the pipettes. In separate trials we determined that there was negligible evaporation of the solution over the course of several hours. Half of the bees were treated with 10,000 freshly harvested *N*. *ceranae* spores in 7 μL of 1.8 M sucrose solution, the rest with pure 1.8 M sucrose solution. After ingestion, we returned bees to the colony of origin. We gathered five bees from each treatment when they were 5 days old (to establish a mature microbiota^26^), 10 days old, and 21 days old, dissected out the midgut and hindgut, froze them in an RNase free microcentrifuge tube at −80 °C, and then delivered the guts in liquid nitrogen to UC Riverside for analyses. From these 270 bees, 148 yielded successful 16S rRNA gene, host Actin qPCR, and *Nosema ceranae* qPCR data (see Table S1 for sample details and raw data).

### Molecular characterization of *Nosema* load and microbiota composition

The midgut and hindgut were transferred to sterile 96 well sample extraction plates from the DNeasy extraction kit (Qiagen, Valencia, CA). To extract DNA from the gut, two sterile 3.2 mm steel beads were added to ~50 µL of 0.1 mm glass beads. To thoroughly lyse the samples, we added 180 µL of Qiagen (Valencia, CA) buffer ATL to each well, and then bead beat the samples for six minutes at 30 hertz with the Tissue Lyser (Qiagen, Valencia, CA). We added 20 µL proteinase K to each sample and incubated them at 56 °C overnight. To complete the extractions, we followed the protocol recommended by the DNeasy Blood and Tissue kit (Qiagen, Valencia CA).

To quantify *N*. *ceranae* infection, we followed a modified version of the qPCR protocol of Bourgeois *et al*.^42^. For each 15 μl reaction we used 7.5 μl of SsoAdvanced Universal SybR Green Supermix (BioRad, Hercules, CA), 3.9 μl Ultrapure water (Invitrogen, Carlsbad, CA), 3 μl of template DNA, and 0.2 μM of each primer. We used the *N*. *ceranae* specific primers NcF (AAGAGTGAGACCTATCAGCTAGTTG) and NcR (CCGTCTCTCAGGCTCCTTCTC). We first used these primers for a standard PCR on a *N*. *ceranae* positive sample and sequenced the PCR product to verify the identity as *N*. *ceranae*. To create a standard curve, we cloned this PCR product using the TopoTA cloning kit (Invitrogen, Carlsbad, CA) and then created serial dilutions of the recombinant clones ranging from 10^8^–10^2^ copies. To quantify the absolute abundance of *N*. *ceranae*, we ran these dilutions on every plate as quantification standards using the CFX96 real time system (BioRad, Hercules, CA). We used the following thermocycler program: 95 °C for 3 minutes followed by 40 cycles of 95 °C for 10 seconds and 58 °C for 30 seconds. Each sample and standard were run in triplicate, including negative controls, and we used the average of the three reactions to determine *N*. *ceranae* load. Amplification efficiency was calculated by the CFX manager software, with all efficiencies ranging between 90–110% and most in the mid 90% range (for an example standard curve, see Fig. S3).

To relativize our *Nosema* qPCR data to a host gene, we performed quantitative PCR with the host housekeeping gene Actin^43^. We used the same reagent mixtures and thermocycler protocol (except with a 57 °C annealing/extension step) as described above with the primers ActinF (TGCCAACACTGTCCTTTCTG) and ActinR (AGAATTGACCCACCAATCCA). For absolute quantification, we used a gBlock custom oligonucleotide spanning the length of the fragment (156 bp, Integrated DNA Technologies, Skokoe, IL) diluted from 10^7^ to 10^3^ copies to create a standard curve. Amplification efficiency was calculated by the CFX manager software, with all efficiencies ranging between 90–110% and most in the mid 90% range (for an example standard curve, see Fig. S2).

To characterize microbiota composition, we used standard protocols to conduct 16S rRNA gene analyses^44^. For PCR reactions we used the plastid-excluding 16S rRNA gene 799F^45^ (CMGGGTATCTAATCCKGTT) and 1115R^46^ (AGGGTTGCGCTCGTTG) indexed primers that amplifies the V5 and V6 variable regions. Briefly, we used a dual barcoding approach with two primers sets to build the Illumina sequencing construct, as in Kembel *et al*.^46^ and McFrederick and Rehan^47^. To sequence these amplicons, we cleaned up the PCR reactions using the PureLink Pro PCR clean up kit (ThermoFisher Scientific, Waltham, MA), normalized each sample to be equimolar with SequalPrep normalization plates (ThermoFisher Scientific, Waltham, MA) and then pooled and sequenced the reactions using a 2 × 300 bp paired end run with V3 reagents on the Illumina MiSeq platform.

### Statistical analyses

To determine the effects of colony, treatment, and days since infection (all fixed, nominal variables) on ranked spore loads, we used Analysis of Variance (ANOVA) and Tukey Honestly Significant Difference (HSD) tests for post-hoc analyses^48^. To identify colonies with low or high infection, we used the Tukey HSD results to identify colonies that significantly differed in intensity of infection.

We used QIIME2 version 2017–11^49^ and the R package vegan^50^ for all other data analyses. For quality control of the 16S rRNA gene data, we viewed quality scores of the DNA sequence and trimmed reads of low-quality regions. We used DADA2^51^ to infer exact sequence variants (ESVs; bacteria that share the exact DNA sequence over the 16S rRNA gene region that we sequenced). To assign taxonomy to each ESV, we trained the Silva database (v. 128^52^) to our primer set in QIIME2, then assigned taxonomy using the sklearn classifier^53^. For alpha and beta diversity analyses, we first aligned representative sequences with MAFFT^54^ and filtered out poorly aligned sections with QIIME’s alignment mask. We built a phylogeny from the resulting alignment using FastTree^55^ and conducted alpha and beta diversity analyses with QIIME2’s core diversity metrics. To determine if the microbiota of *N*. *ceranae* treated versus control bees differed, we performed Permutational MANOVA (PerMANOVA) and Non-metric Multidimensional Scaling (NMDS) analyses in vegan.

To detect differences in the microbiota, we ran several different Permutational MANOVA analyses in vegan, all with Bray-Curtis distances as the dependent variables. First, we used treatment, the day post-infection, and the interaction between treatment and day post-infection as explanatory variables, and colony of origin as strata. Next, we used colony infection level, day post infection, and the interaction between colony infection level and day post infection as explanatory variables with colony of origin as strata. To understand whether treatment affected the results of the colony infection level test, we split the data set into two: one dataset with control bees only and one with treated bees only. We then ran separate Permutational MANOVA analyses with colony infection level, day post infection, and the interaction between colony infection level and day post infection as explanatory variables with colony of origin as strata. To check for differences in dispersion between groups, we used vegan’s betadisper function.

To understand whether *N*. *ceranae* infection was associated with increased or decreased abundance of specific bacteria, we tested for differential abundance of specific ESVs using an Ancom analysis^56^ in QIIME2 (False Discovery Rate adjusted P < 0.05, N = 144). We additionally performed separate Ancom analysis on feature tables broken out for control bees only (N = 68) or *Nosema* treated bees only (N = 76).

## Supplementary information

Supplementary Info Titlepage and Figs

Table S1.

## Data Availability

Raw 16S rRNA gene sequences are freely available from NCBI’s Sequence Read Archive (SRA accession number SRP144635). All other data are available in Supplemental Table S1.
