# Supplementary material for: Intensity of Nosema ceranae infection is associated with specific honey bee gut bacteria and weakly associated with gut microbiome structure
Source: Sci Rep. 2019 Mar 7;9:3820. doi: 10.1038/s41598-019-40347-6 (PMC6405881; doi:10.1038/s41598-019-40347-6)
Supplement: Supplementary file 1 — Supplementary Info Titlepage and Figs [file 41598_2019_40347_MOESM1_ESM.pdf]

SUPPLEMENTARY INFORMATION FOR:

Intensity of *Nosema ceranae* infection is associated with specific honey bee gut bacteria and weakly associated with overall gut microbiome structure

Andrey Rubanov[1], Kaleigh A. Russell[2], Jason A. Rothman[3], James C. Nieh[1*], and Quinn S. McFrederick[2,3*]

*Corresponding authors:

jnieh@ucsd.edu

quinn.mcfrederick@ucr.edu

[1]UCSD
Division of Biological Sciences
Section of Ecology, Behavior, and Evolution
9500 Gilman Drive, MC0116
La Jolla, CA 92093-0116
USA

[2]University of California, Riverside
Department of Entomology
900 University Avenue
Riverside, CA 92521

[3]University of California, Riverside
Microbiology Graduate Program
900 University Avenue
Riverside, CA 92521

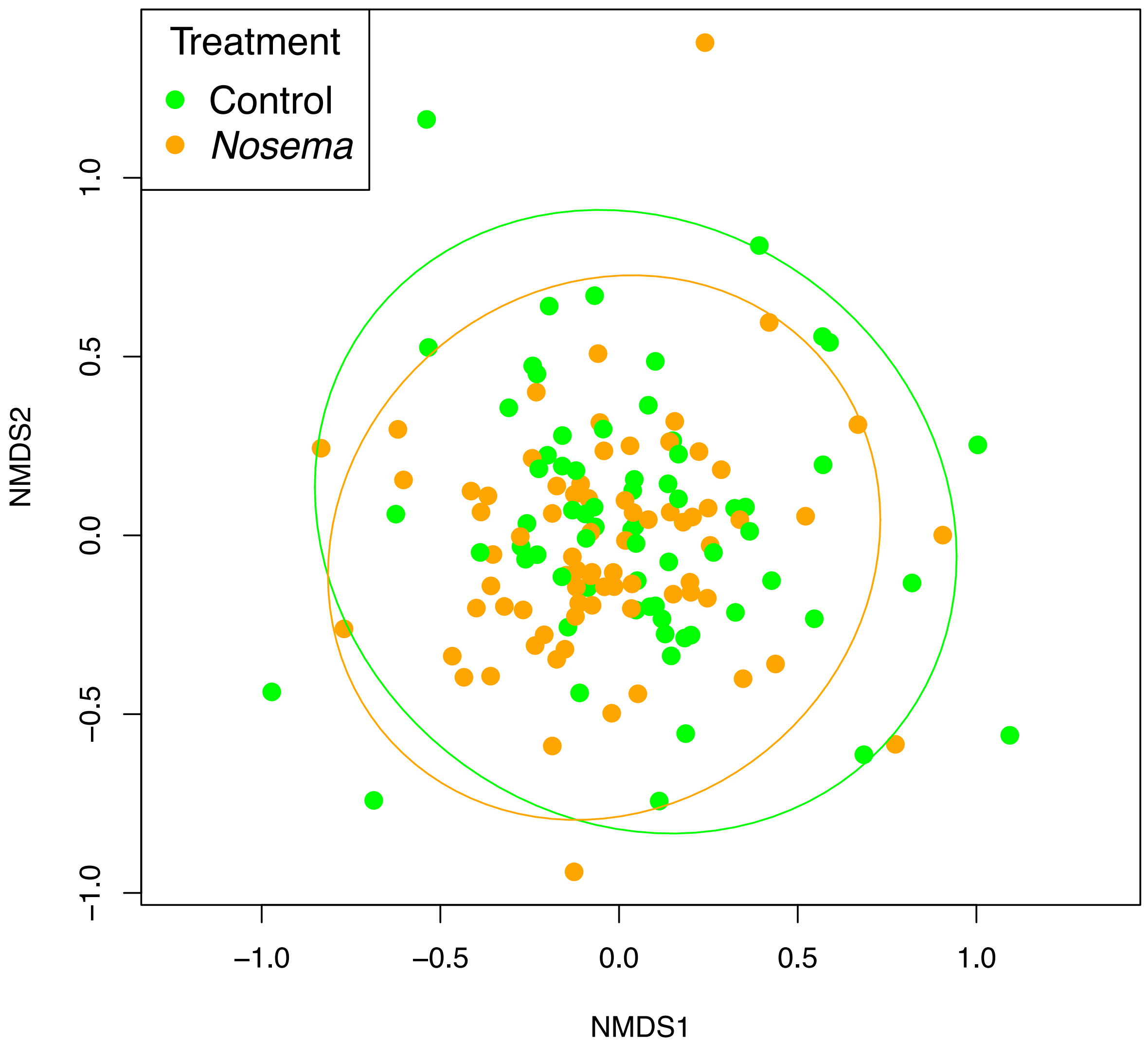

Fig. S1. NMDS ordination of Bray-Curtis distances of gut microbiota composition showed no significant difference in control versus infected bees (Adonis $F_{1,142} = 0.91$, $P = 0.56$).

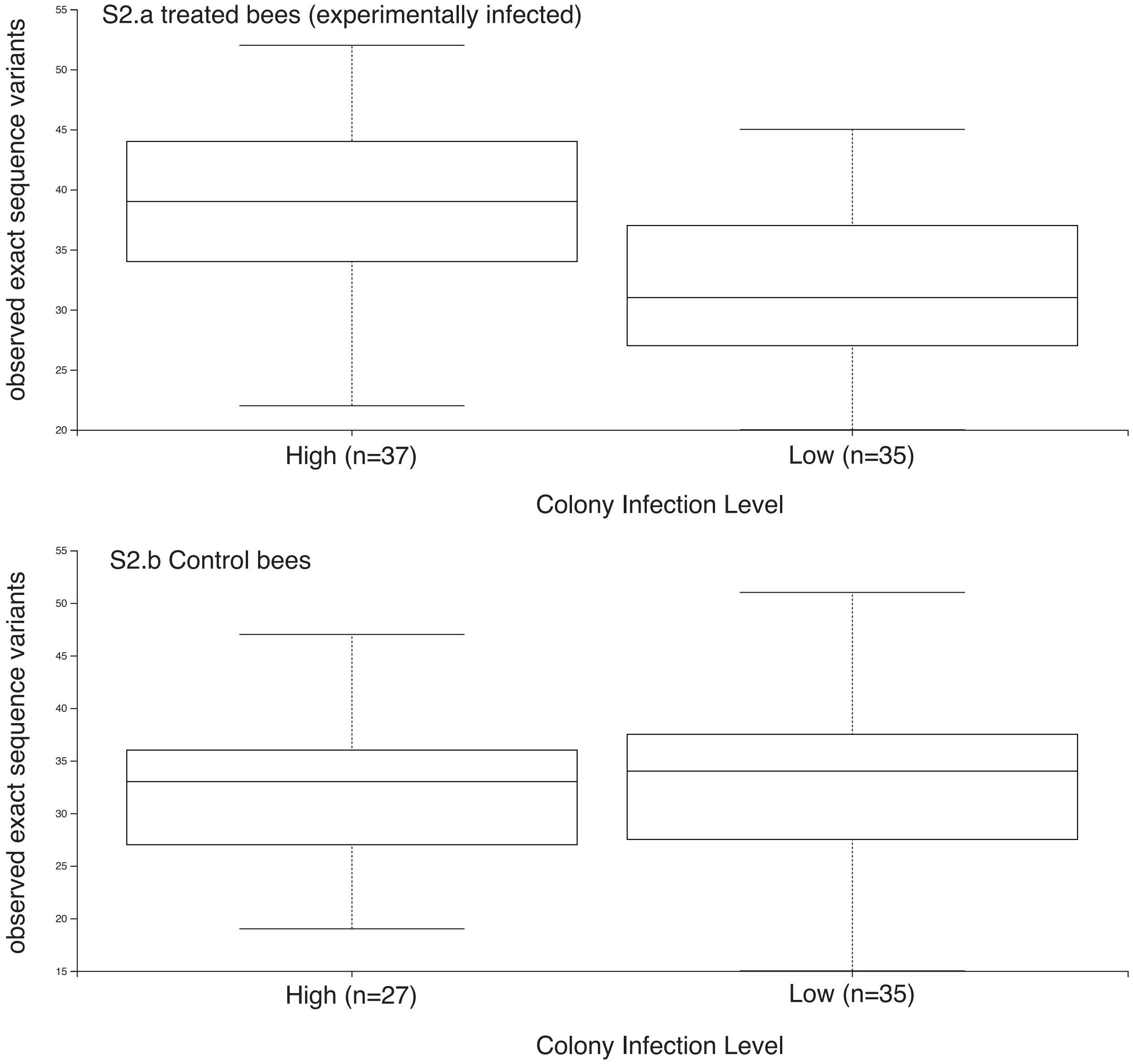

Fig. S2. For *Nosema* treated samples, high infection level colonies exhibited more ESVs per sample compared to low infection colonies (Fig. S2.a, Kruskal-Wallis H_{71} = 12.07, $P < 0.0006$). For control bees alpha diversity did not differ between high and low infection level colonies (Fig. S2.b, Kruskal-Wallis H_{64} = 0.12, $P = 0.75$).

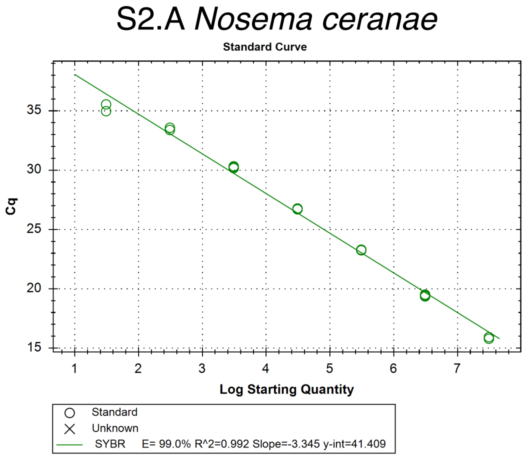

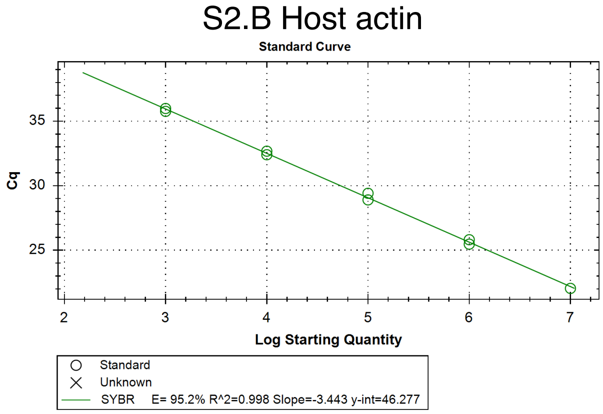

Fig. S3. Example standard curves showing qPCR amplification efficiency (calculated by the BioRad CFX manager software). All efficiencies ranged between 90-110% with most in the mid 90% range.
